# A dataset of global ocean alkaline phosphatase activity

**DOI:** 10.1038/s41597-023-02081-7

**Published:** 2023-04-13

**Authors:** Bei Su, Xianrui Song, Solange Duhamel, Claire Mahaffey, Clare Davis, Ingrid Ivančić, Jihua Liu

**Affiliations:** 1 Institute of Marine Science and Technology, Shandong University, Qingdao, Shandong 266237 China; 2 Southern Marine Science and Engineering Guangdong Laboratory, Zhuhai, China; 3 Department of Molecular and Cellular Biology, University of Arizona, Tucson, AZ USA; 4 Department of Earth, Ocean and Ecological Sciences, University of Liverpool, Merseyside, UK; 5 Now at Springer Nature, London, UK; 6 Center for Marine Research, Ruđer Bošković Institute, G. Paliaga 5, HR-52210 Rovinj, Croatia

**Keywords:** Ocean sciences, Element cycles

## Abstract

Utilisation of dissolved organic phosphorus (DOP) by marine microbes as an alternative phosphorus (P) source when phosphate is scarce can help sustain non-Redfieldian carbon:nitrogen:phosphorus ratios and efficient ocean carbon export. However, global spatial patterns and rates of microbial DOP utilisation are poorly investigated. Alkaline phosphatase (AP) is an important enzyme group that facilitates the remineralisation of DOP to phosphate and thus its activity is a good proxy for DOP-utilisation, particularly in P-stressed regions. We present a Global Alkaline Phosphatase Activity Dataset (GAPAD) with 4083 measurements collected from 79 published manuscripts and one database. Measurements are organised into four groups based on substrate and further subdivided into seven size fractions based on filtration pore size. The dataset is globally distributed and covers major oceanic regions, with most measurements collected in the upper 20 m of low-latitude oceanic regions during summer since 1997. This dataset can help support future studies assessing global ocean P supply from DOP utilisation and provide a useful data reference for both field investigations and modelling activities.

## Background & Summary

Phosphorus (P) is an essential element for marine life^1^ and the ultimate limiting nutrient of ocean productivity^2^. Dissolved inorganic phosphorus (DIP), essentially phosphate, is the preferred P source for most microorganisms, but is often scarce in the surface ocean, especially in the North Atlantic Subtropical Gyre and the Mediterranean Sea^3–5^. Dissolved organic phosphorus (DOP) comprises the majority of the dissolved P pool in the surface open ocean, but is not readily available to many microorganisms^6^. Alkaline phosphatase (AP), a group of metalloenzymes that catalyses the hydrolysis of a broad spectrum of marine DOP compounds, enables remineralisation of DOP to DIP^7,8^ and therefore provides the potential to alleviate phosphorus limitation for marine organisms.

**Fig. 1:**
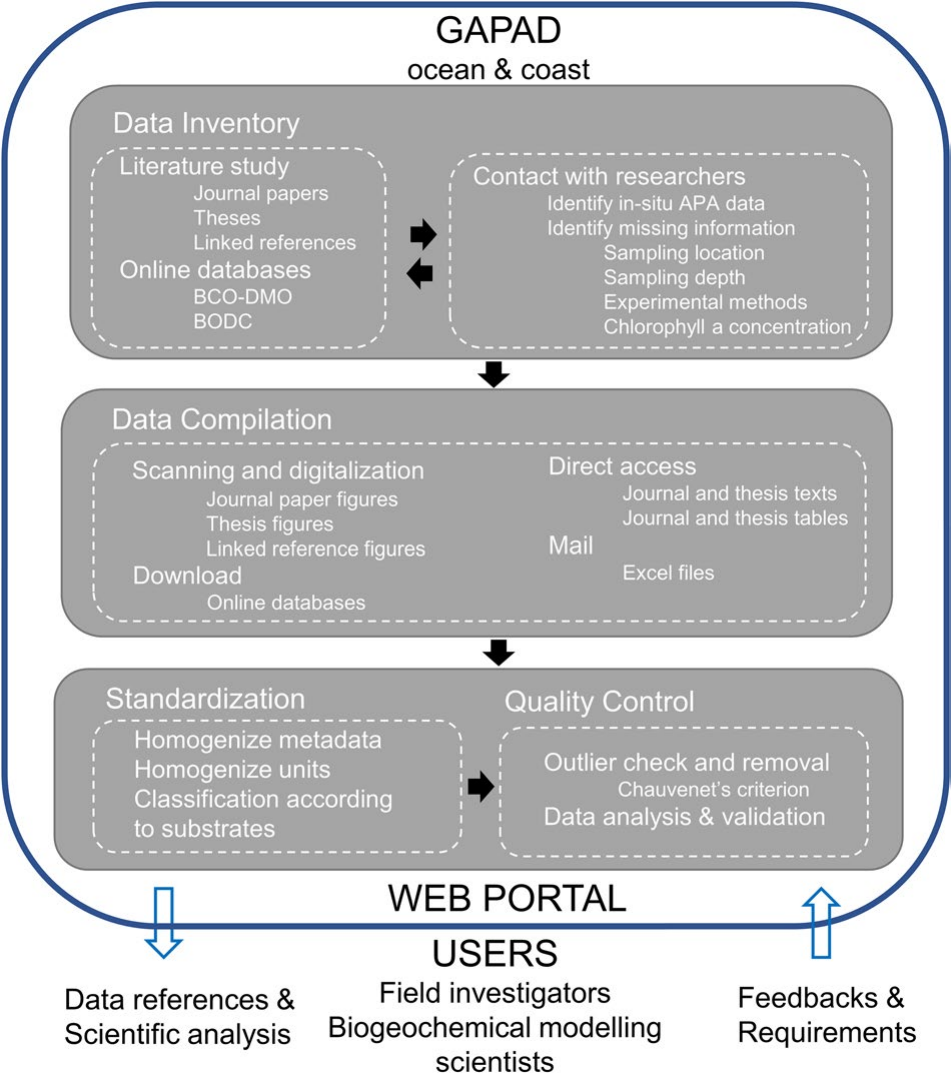
Work-flow of GAPAD compilation, standardization and quality-control.

Alkaline phosphatase is often induced at extremely low phosphate concentrations, i.e., below a threshold phosphate concentration of ~30 nmol L^−1^ ^9^, resulting in a high rate of alkaline phosphatase activity (APA) in P-limited oceanic regions^10,11^. Therefore, APA is an important indicator of P-limitation and a useful proxy to gauge DOP-utilisation by marine microorganisms^12^. Studies quantifying APA started in the 1970s^13^ and have greatly improved our understanding of the marine phosphorus cycle. To facilitate better understanding of the role of AP in P supply via microbial DOP-utilisation, we present a Global Alkaline Phosphatase Activity Dataset (GAPAD) including 4083 measurements during the last 50 years, with 4051 measurements from 79 published manuscripts and 32 measurements from 1 database^14^. Global Alkaline Phosphatase Activity Dataset is the most comprehensive dataset published thus far since it includes not only APA measurements from the global tropical and subtropical oceans, but also their temporal and spatial information, as well as relevant environmental parameters including dissolved inorganic and organic phosphorus concentrations, chlorophyll a concentration, salinity and temperature^14^. The workflow of the GAPAD compilation is shown in Fig. 1.

**Fig. 2:**
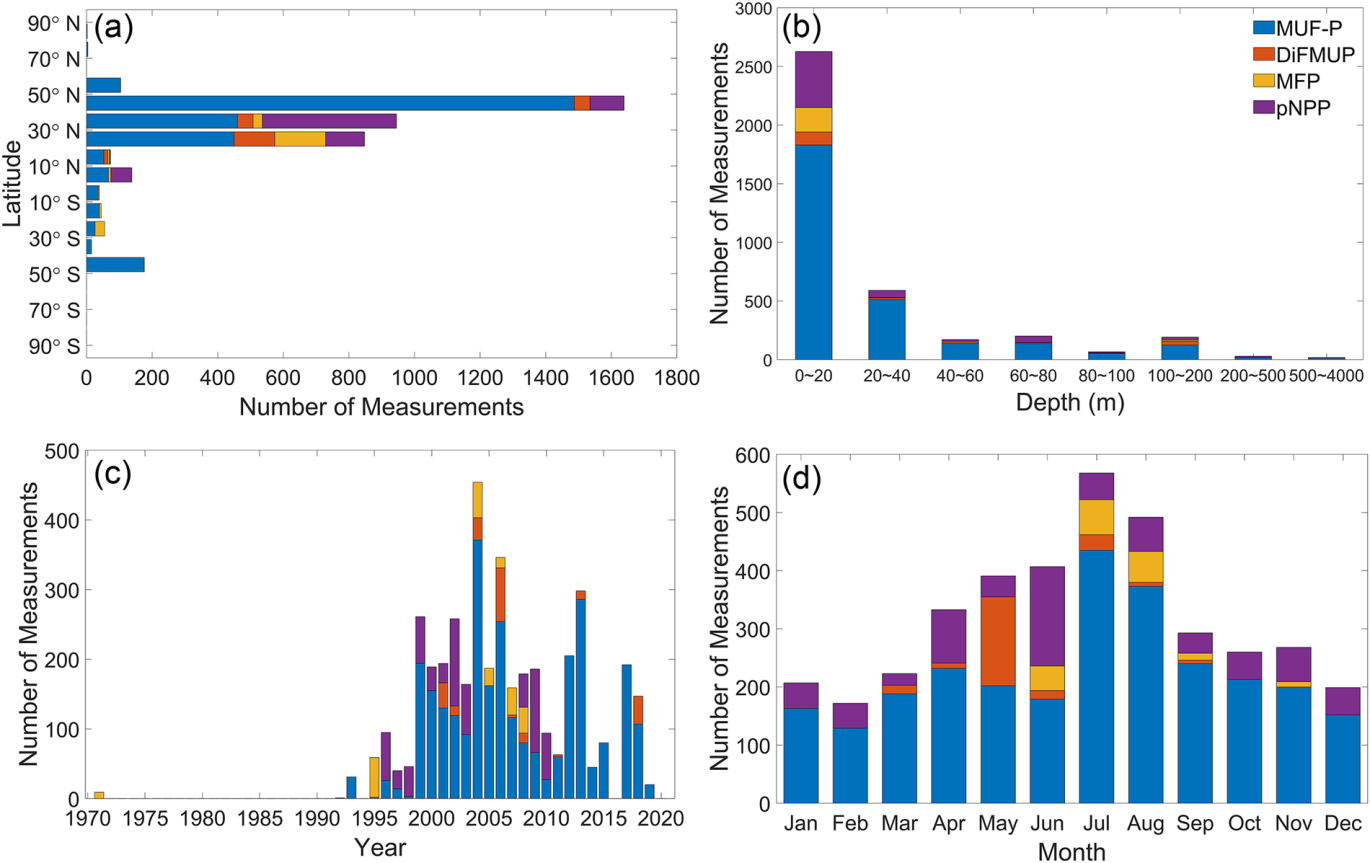
APA measurement distributions in the ocean. (**a**) Latitudinal, (**b**) Vertical, (**c**) Yearly, and (**d**) Monthly distributions of APA measurements with each substrate colored. Blue, orange, yellow and purple bars represent measurements with the substrates MUF-P, DiFMUP, MFP and pNPP, respectively.

Four substrates have been used to measure APA in GAPAD, i.e., 4-methylumbelliferyl phosphate (MUF-P), 6, 8-difluoro-4-methylumbelliferylphosphate (DiFMUP), 3-O-methylfluorescein phosphate (MFP), and paranitrophenyl phosphate (pNPP) (Fig. 2). There are respectively 2919, 232, 233 and 699 measurements collected from 54, 10, 6 and 9 studies applying MUF-P, DiFMUP, MFP and pNPP as substrates^14^. Although minor differences exist in their experimental methods, rates are often measured at saturating substrate concentrations to obtain the potential activity^15^, except when concentrations between 30 and 100 nmol L^−1^ are used to represent *in-situ* substrate concentration^16–19^. Furthermore, we have applied statistical methods to flag outliers in order to improve the quality of the dataset (Table 1). The majority of the APA measurements are within a latitudinal span of 50°S–50°N, with a higher density in the northern hemisphere (Fig. 2a). The sampling depths range from 0 to 4000 m, with most sampling depths located within 20 m of the surface (Fig. 2b). Measurements were performed between years 1971 and 2019 (Fig. 2c), and there are more measurements in summer months (400–600 per month) compared to winter months (~200 per month; Fig. 2d).

**Table 1:** Results of the outlier identification method applying to the substrate MUF-P. Definitions of the different fractions of APA in this table are described in the Methods section.

APA	Number of measurements	Number of identified outliers	${\bar{{\boldsymbol{x}}}}_{{\boldsymbol{l}}{\boldsymbol{o}}{\boldsymbol{g}}}$ before/after outlier identification (nmol L^−1^ h^−1^)	*s*_*log*_ before/after outlier identification (nmol L^−1^ h^−1^)
Bulk	2266	0	0.89/0.89	1.19/1.19
Dissolved	292	0	0.49/0.49	1.21/1.21
Particulate	116	2	0.0045/−0.047	0.97/0.89
Bacterial	92	0	1.31/1.31	0.77/0.77
Phytoplankton	93	0	1.78/1.78	0.76/0.76
*Trichodesmium*	30	0	0.37/0.37	1.67/1.67

${\bar{{\boldsymbol{x}}}}_{{\boldsymbol{l}}{\boldsymbol{o}}{\boldsymbol{g}}}$: log10-transformed mean values.

***s***_***log***_ : log10-transformed standard deviation values.

**Fig. 3:**
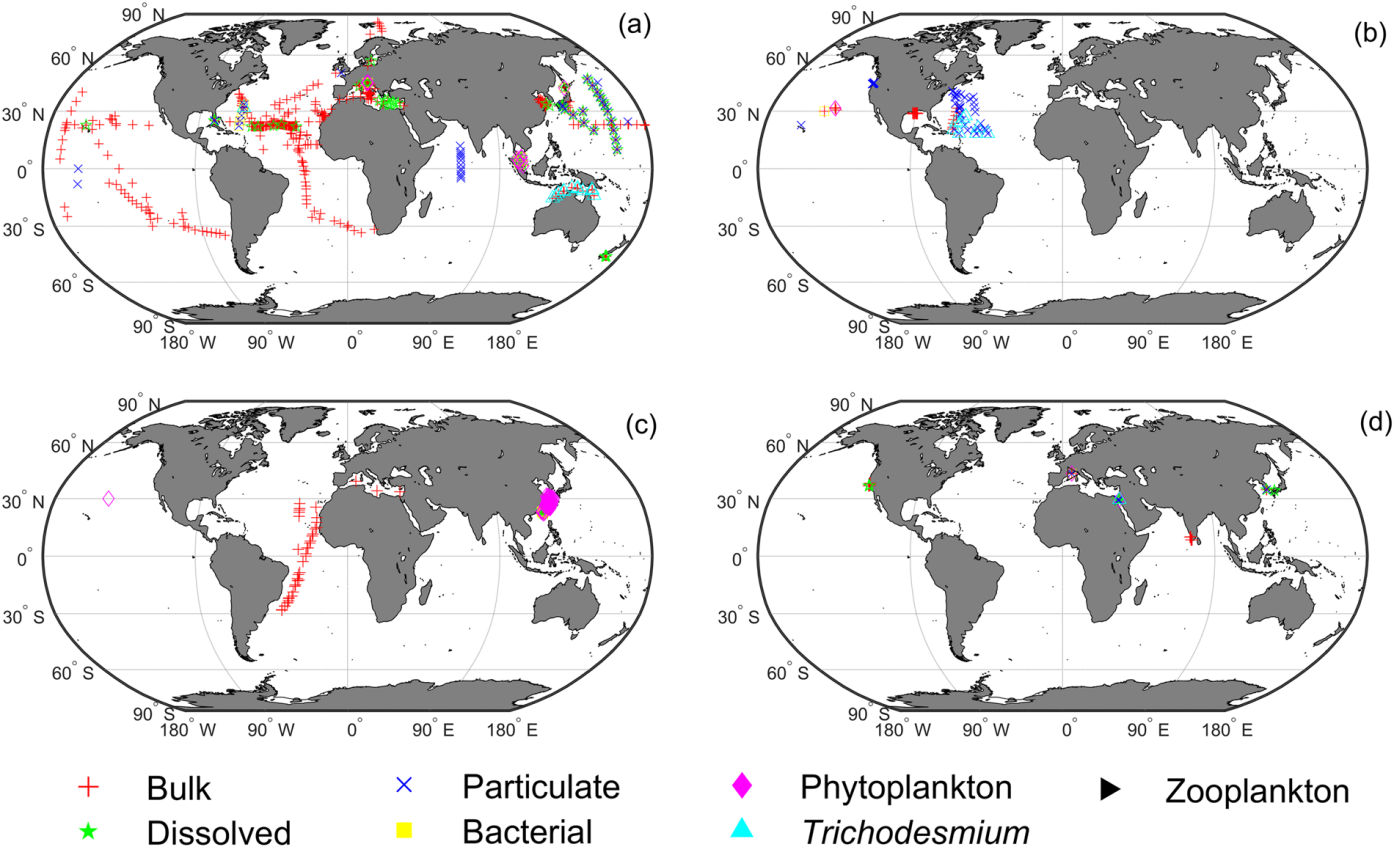
Global distribution of APA measurements in GAPAD for the different four substrates. (**a**) MUF-P, (**b**) DiFMUP, (**c**) MFP and (**d**) pNPP. Each marker represents a fraction, with red plus, green pentagram, blue cross, yellow square, magenta diamond, cyan upward-pointing triangle and black right-pointing triangle representing bulk APA, dissolved APA, particulate APA, bacterial APA, phytoplankton APA, *Trichodesmium* APA, and zooplankton APA respectively. Definitions of the different fractions of APA in this figure are described in the Methods section.

**Fig. 4:**
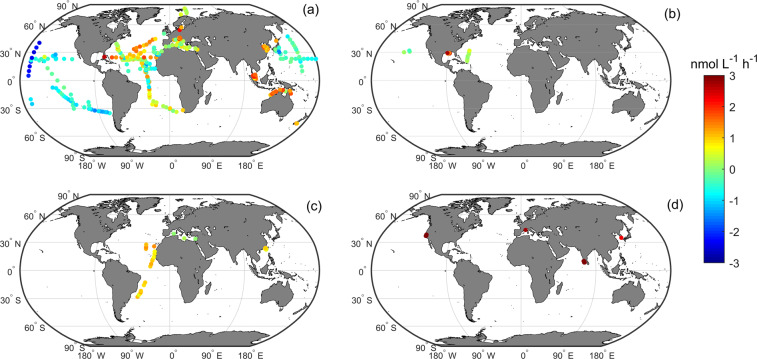
Global distribution of Log10-transformed full-depth-averaged bulk APA measurements in GAPAD for the four different substrates. (**a**) MUF-P, (**b**) DiFMUP, (**c**) MFP and (d) pNPP.

Alkaline phosphatase activity measured with the substrate MUF-P is the most common and widely distributed in global oceans (Fig. 3a). In the North Atlantic and the Northeast Pacific oceans, APA was measured with the substrate DiFMUP, with fractions of particulate APA, phytoplankton APA, and *Trichodesmium* APA mostly measured in the North Atlantic (Fig. 3b). Average bulk APA (APA measured with unfiltered water) rates in the North Atlantic (2.49 ± 2.34 nmol L^−1^ h^−1^, n = 77, mean ± SD) are higher than in the eastern Pacific (0.84 ± 0.38 nmol L^−1^ h^−1^, n = 4; Fig. 4b). For the MFP substrate, bulk APA rates are available in the Mediterranean Sea and the Atlantic, while phytoplankton APA was only measured in the East China Sea (Fig. 3c). All APA measurements with pNPP as the substrate are from coastal waters of the Pacific, the Indian Ocean and the Mediterranean Sea (Fig. 3d).

**Fig. 5:**
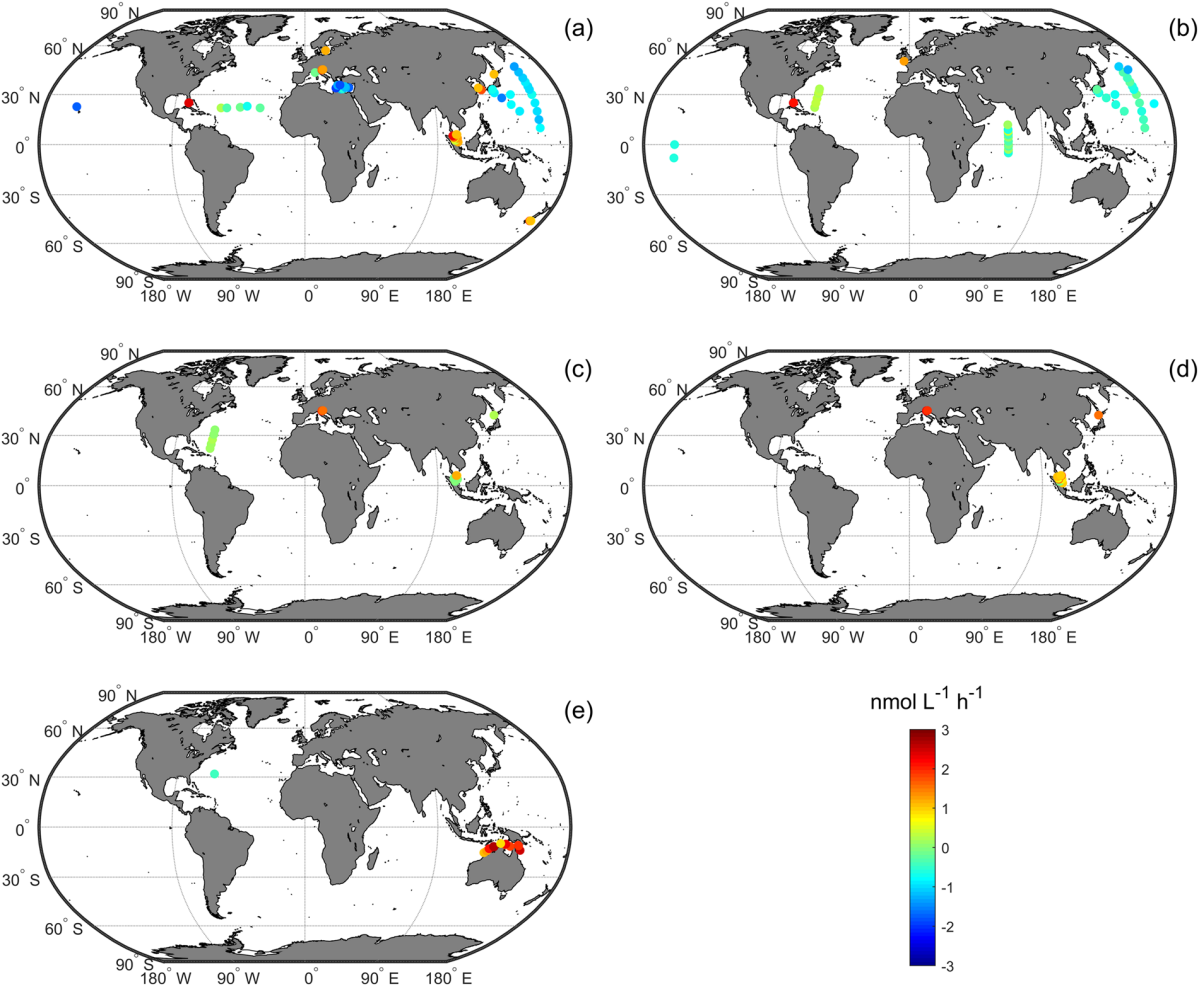
Global distribution of Log10-transformed full-depth-averaged APA measurement of MUF-P in GAPAD for the five different fractions. (**a**) dissolved APA, (**b**) particulate APA, (**c**) bacterial APA, (**d**) phytoplankton APA and (**e**) *Trichodesmium* APA.

We have also divided APA measured with each substrate according to different fraction types, i.e., bulk APA, dissolved APA, particulate APA, bacterial APA, phytoplankton APA, *Trichodesmium* APA, and zooplankton APA (Fig. 3). Since APA measured with the substrate MUF-P is the most abundant and widely distributed in GAPAD (Fig. 3a), with bulk APA covering a large part of the Atlantic, the Pacific, and the Mediterranean Sea, we further analyse their distributions and rates (Figs. 4a, 5). The bulk APA rates near the coasts (161.96 ± 523.03 nmol L^−1^ h^−1^, n = 1528, defined as water depth less than 1000m in this study) are generally higher than those in the open ocean (2.60 ± 6.94 nmol L^−1^ h^−1^, n = 749,  defined as water depth>1000m). The highest APA rate (6583 nmol L^−1^ h^−1^) is in the northern Adriatic Sea (Fig. 4a). Dissolved APA have been measured in the Northwest Pacific, the Mediterranean Sea, and the North Atlantic (Fig. 5a), whereas particulate APA has been measured in the Northwest Pacific, the Equatorial west Atlantic and the Indian Ocean (Fig. 5b). Bacterial APA has been measured in the North Atlantic and the South China Sea (Fig. 5c), whereas the phytoplankton APA has also been measured mainly in the South China Sea, Adriatic Sea and Bay of Biscay (Fig. 5d) and *Trichodesmium* APA has been measured in the North Atlantic and near the coast of northern Australia in GAPAD (Fig. 5e).

In oligotrophic marine environments, AP may contribute a large fraction of DOP utilisation and is therefore important for supporting the non-Redfieldian carbon:nitrogen:phosphorus (C:N:P) ratios of marine organisms and marine carbon export^20^. Due to the important role of AP in alleviating P-limitation for diazotrophs and supporting N_2_ fixation, it may also control ecological diversity by giving them an ecological advantage when competing for resources with non-diazotrophs^9,20–24^. Global Alkaline Phosphatase Activity Dataset will provide a new resource for the study of the global ocean phosphorus cycling, further elucidating impacts on these critical processes.

## Methods

Four substrates have been used in the APA measurements, i.e., MUF-P^8,9,15–19,25–72^, DiFMUP^10,12,73–80^, MFP^13,81–85^ and pNPP^86–94^. Since a fluorescent (or colored) product is released when a substrate is hydrolysed by AP in a seawater sample, APA can be measured by detecting the changes in fluorescence (or color) over time. Measurements were mostly carried out with unfiltered water (bulk APA) and two pre-filtrations with filter sizes of 0.22 µm and 3 µm (size-fractionated APA). The dissolved fraction is often identified as <0.22 µm, even though this might contain nanoparticles, colloidal nanogels and/or viruses^95^. The particulate fraction is usually identified as >0.22 µm^30^, except in a few studies in this compilation^75,87,88^, using >0.25 µm or >0.4 µm. The bacterial fraction, containing heterotrophic bacteria and picocyanobacteria, is often identified as 0.22–3 µm^40^, except Duhamel *et al*., Lim *et al*. and Bogé *et al*., who used 0.2–0.6 µm^12^ or 0.2–0.8 µm^36^, 0.2–2 µm^45^ and 0.25–5 µm^87^, respectively. The phytoplankton fraction is often from samples prefiltered through meshes of different pore sizes, e.g., 120 µm^83^, 200 µm^41^ and 1 mm^81,82^ to remove zooplankton. Lim *et al*. and Bogé *et al*. identify the phytoplankton fraction as 2–20 µm^45^ and 5–90 µm^87^, respectively. Several studies also identify a *Trichodesmium* fraction^49,51,76,94,96,97^ and a zooplankton fraction (>90 µm)^86–88^.

For samples collected on filters with different pore sizes, samples are usually re-suspended in sterile phosphate-free artificial seawater^10^ or autoclaved pre-filtered seawater^47^ for several minutes before the start of the experiment. Standard fluorescent products, e.g., MUF (methylumbelliferone), with concentrations typically ranging from 0 to 2000 nM are used to produce the standard curve for converting the rate of change in fluorescence to a substrate hydrolysis rate^32^. Fluorescence is measured using a fluorometer immediately after substrate addition and at regular intervals (e.g., 30 min). The rate of APA is derived from the changes of fluorescence over time and converted to hydrolysis rate using the calibration curve. To improve the accuracy of the calculation, seawater blanks, boiled samples or ultrapure water are used to correct fluorescence measurements and account for abiotic substrate hydrolysis or degradation^12,32,35^. Enzyme-kinetic parameters (Michaelis-Menten parameters including the maximum hydrolysis rate (Vmax), and the half-saturation constant (Km)) are also determined in some studies using data from incubations of different substrate concentrations in unfiltered seawater^35^.

The APA data have been collected by searching published manuscripts with key words ‘alkaline phosphatase; alkaline phosphatase activity; AP; APA; ocean; coast’ in multiple academic service platforms, i.e., the Web of Science (https://www.webofscience.com/), the China National Knowledge Infrastructure (CNKI, https://www.cnki.net/), and the Wanfang Data Knowledge Service Platform (https://www.wanfangdata.com.cn/), as well as available databases, i.e., the Biological & Chemical Oceanography Data Management Office (BCO-DMO) and the British Oceanographic Data Centre (BODC). We reported APA measurements in environmental samples and combined all available measurements to create the most comprehensive global coverage of *in-situ* APA with the procedures described in Fig. 1. Most data have been obtained directly from the figures and tables in the published manuscripts. Data that could not be obtained directly have been digitized from figures using the Engauge Digitizer 12.1 software or provided by the authors on request^25,28,33,37–41,46,47,54,55,82,98,99^. Some authors provided unpublished data from their dissertations^100–102^, which are then included in GAPAD. Data presented in appendices of published manuscripts are also included in this compilation^11^.

The units of APA are often reported as volumetric rates, e.g., nmol L^−1^ h^−1^, µmol L^−1^ h^−1^, or nmol L^−1^ min^−1^. However, some APA measurements are normalized to other parameters, e.g., chlorophyll a concentration (pmol µg Chl^−1^ min^−1^)^81,82^, cell abundance (nmol cell^−1^ h^−1^)^12^ or *Trichodesmium* colony abundance (nmol colony^−1^ h^−1^)^51^. We unified the units to the volumetric rates by multiplying them by the *in-situ* concentrations of the respective parameters. Finally, we transformed all units to nmol L^−1^ h^−1^.

## Data Records

Global Alkaline Phosphatase Activity Dataset is included in 4 sheets of a dataset file according to substrate type, i.e., MUF-P, DiFMUP, MFP, and pNPP. Each sheet includes the following fields for each record:

Source of data

Latitude (−90° to 90°)

Longitude (−180° to 180°)

Sampling depth (m)

Cruise

Site/Station

Year

Month

APA (nmol L^−1^ h^−1^)

Bulk

Dissolved

Particulate

Bacteria

Phytoplankton

*Trichodesmium*

Zooplankton

Dissolved Inorganic Nitrogen (nmol L^−1^)

Dissolved Inorganic Phosphorus (nmol L^−1^)

Dissolved Organic Phosphorus (nmol L^−1^)

Chlorophyll a (µg L^−1^)

Colony abundance (colony L^−1^)

Cell abundance (cell L^−1^)

Salinity (psu)

Temperature (°C)

Alkaline phosphatase activity measurements are subdivided into seven fractions according to their filtration sizes as outlined in the Methods section described above. In addition, environmental parameters reported to potentially impact rates of APA are also included whenever they are available in published articles or databases, and a summary of detailed sources of APA data is on sheet 5 of the dataset file. The dataset file in Excel Workbook (xlsx) format can be accessed on Figshare using the link (10.6084/m9.figshare.c.6340244.v1)^14^. ‘– 999’ denotes missing data. The dataset will be updated by the authors when new data are available.

## Technical Validation

Alkaline phosphatase activity in the ocean ranges from below the detection limit (denoted by 0, e.g. <=0.002 nmol L^-1^ h^-1^ in Yamaguichi *et al*.^68^) to very high rates as much as 6583 nmol L^−1^ h^−1^ for MUF-P, which is largely controlled by ambient DIP concentration and DOP availability^9,36,44^. Therefore, APA rates are not normally distributed and show a positively skewed distribution with long tails of high values. However, the collected APA rates are approximately log-normally distributed after excluding the data points of zero.

In order to control the quality of GAPAD, we applied the Chauvenet’s criterion to identify suspicious outliers whose probability of deviation from the mean is less than 1/(2n)^103^, where n is the number of measurements. Since the APA rates are approximately log-normal distributed, the method is only applied to the log-transformed non-zero data. We use the MATLAB *norminv* function to calculate the critical value (x_log_*) with the mean ${\bar{x}}_{log}$, the standard deviation *s*_*log*_, and the evaluated probability values in *p*, where *p* is calculated from 1-1/(4n) instead of 1/(2n), because the Chauvenet’s criterion is a two-tailed test and only data at the tail with high values will be identified. Then data points with values larger than the critical value x_log_* will be flagged. In this study, we apply the method only once in each of the seven fractions of the four groups categorized by substrate respectively, except when it has less than 20 measurements.

We accept all the data which are not flagged by the Chauvenet’s criterion. For the flagged suspicious outliers, we determine whether to exclude them from GAPAD or not after carefully assessing their values to validate that they are very skewed from the approximate log-normal distribution. The results of the quality control applied following this approach are shown in Table 1.

## Usage Note

Global alkaline phosphatase activity dataset can serve as a reference to field investigators for assessing their results, and to biogeochemical modelling scientists for model validation. With our APA dataset, the role of environmental factors affecting APA can also be examined to understand the role of global ocean phosphate supply from AP-catalysed DOP utilisation in response to future climate change.

## Supplementary information

Dataset 1

## Data Availability

The source codes for identifying outliers used in this paper are available at https://github.com/BGM-USD2020/GAPAD_codes.git.
